# High-Efficiency, Two-Step Scarless–Markerless Genome Genetic Modification in *Salmonella enterica*

**DOI:** 10.1007/s00284-016-1002-3

**Published:** 2016-02-16

**Authors:** Shizhong Geng, Qin Tian, Shuming An, Zhiming Pan, Xiang Chen, Xinan Jiao

**Affiliations:** Jiangsu Key Laboratory of Zoonosis, Jiangsu Co-Innovation Center for Prevention and Control of Important Animal Infectious Diseases and Zoonoses, Yangzhou University, Yangzhou, 225009 China

## Abstract

We present a two-step method for scarless–markerless genome genetic modification in *Salmonella enterica* based on the improved suicide plasmid pGMB152. The whole *LacZYA* gene can provide a *lacZ*-based blue/white screening strategy for fast selection of double-crossover mutants by allelic exchange. The high efficiency of this genetic engineering strategy permits the study of gene function by gene knockin, site-directed mutagenesis, and gene knockout to construct live attenuated vaccines.

## Introduction

Genome genetic modification, including gene knockout, gene knockin, and site-directed mutagenesis by replacing target genes with in vitro-modified alleles is an essential tool for studying the genetic basis of bacterial phenotypes at the molecular level [4, 17]. Current methods are largely based on the principles of allelic exchange using a conventional suicide plasmid, such as pGMB151 [8, 11]. pGMB151 is a derivative of the suicide plasmid pKNG101 [11] and includes *oriR6K*, *RP4*, *sacBR*, *insB,* and *strAB* with the *bla* gene from plasmid pBR322 cloned at the *Xba* I site. It is a suicide plasmid because of its requirement for the pir protein, which is supplied by λ*pir* in certain strains of *Escherichia coli*, such as SM10 [18].

The current suicide plasmid method may not be very efficient because of low recombination rates or illegitimate recombination, so they are often improved [12, 17]. To improve the efficiency of selecting precise gene-deletion mutants and simplifying the process of genome genetic modification, the *sacB* gene was introduced into a suicide plasmid for a kind of counterselection. The *sacB* gene in the suicide plasmid encodes levansucrase, which hydrolyzes sucrose and glucose to fructose, resulting in the production of a toxic fructan that kills the recombinants containing the suicide plasmid. Thus, sucrose is often used to screen bacteria that have lost the suicide plasmid after a double-crossover event in a single-crossover recombinant. Loss of the suicide plasmid can be easily verified via loss of vector-encoded antibiotic resistance. However, this kind of sucrose counterselection only offers one possibility, and its susceptibility to sucrose is variable in different kinds of gram-negative bacteria: for example, in *Salmonellaenterica*, most single-crossover recombinant bacteria show resistance to sucrose, and a few single-crossover recombinant bacteria are sensitive to sucrose [7, 16].

Additionally, antibiotic resistance genes have been used to replace the target gene as selectable markers of successful gene deletion [8, 14, 15, 17]. Although some enzymes may also remove the antibiotic resistance gene, its flanking sequences may not be completely removed. This is an undesirable situation, particularly when precise and scarless–markerless genome genetic modifications are required, such as in genetically engineered attenuated vaccines.

A precise and scarless–markerless genome genetic modification, such as gene knockout for attenuated *Salmonella* vaccines, gene knockin, and site-directed mutagenesis are more difficult without any exogenous nucleotide residuals. The main problem is that double-crossover events following the loss of a suicide plasmid are very difficult to distinguish because of the low frequency of allelic exchange and resistance to sucrose. We believe that a suicide plasmid should have another visual biomarker as a counter selectable tool. Thus, in this study, to improve the efficiency of allelic exchange for genome genetic modification, a novel pGMB152 vector was constructed with the *lacZYA* operon incorporated into pGMB151 to provide *LacZ*-based blue–white selection [1]. The *LacZ* gene, a well-known reporter gene expressing β-galactosidase, can provide blue–white selection to make the existence of molecular cloning visible [2, 6, 13] and could be used as a biomarker for the suicide plasmid. Bacteria colonies with pGMB152 will show as blue and those without pGMB152 will show as white; therefore, bacterial colonies with different colors could be distinguished easily during the different processes of genome genetic modification. The new plasmid was used to knock out the *hsdM* gene [9] in *Salmonellapullorum* to construct a candidate-attenuated *Salmonella* vaccine, to repair pseudogene *flhA* by knockin and pseudogene *flgk* by site-directed mutagenesis.

## Materials and Methods

### Strains, Plasmids, and Media

 The strains and plasmids used in this study are listed in Table 1. Strains were grown routinely in rich liquid or solid Luria Broth media (LB). The media was supplemented with kanamycin (50 μg/mL), ampicillin (100 μg/mL), and streptomycin (25 μg/mL) as required. Solid LB media with 10 % sucrose and without NaCl was used to screen for gene-deletion mutants during the process of allelic exchange.

**Table 1:** Strains and plasmids used in this study

Material	Name	Use	Source	Reference
Bacteria	*S.*Pullorum S06004	Recipient strain	Our laboratory	[9]
	*E*.*coli*. χ7213	Donor strain	Gift from Dr. R. Curtiss III	[10]
	*E*.*coli*.spy372	For cloning	Gift from Dr. R. Curtiss III	
	*E*.*coli*. DH5α	For cloning	Takara company	
Plasmids	pGMB151	Suicide plasmid	Our laboratory	[11]
	pGMB152	Suicide plasmid	This study	
	pFUSE	To offer *Lac*ZYA gene	Our laboratory	[1]
	pMD20-T	For cloning	Takara company	

### Construction of Suicide Plasmid pGMB152

*The lacZYA* cassette was excised from pFUSE (Deng et al. [4]) using restriction endonucleases *Sam* I and *Sal* I and ligated into pGMB151 digested with the same restriction recognition sites. This novel plasmid was named pGMB152.

### Two-Step Scarless–Markerless Knockout of *hsdM* Gene

The chloramphenicol resistance gene (*Cm*^*R*^) from plasmid pKD3, with 54 bp of homologous sequences of the *hsdM* gene flanking the two ends of *Cm*^*R*^, was PCR amplified using primers *hsdM*-*λ*-F/R. This fragment was transformed into *S. pullorum* S06004 to inactivate the *hsdM* gene, with the help of plasmid pKD46 by the λ-red method, to construct *S. Pullorum hsdM::Cm*^*R*^ with an antibiotic marker [3].

An in vitro-modified allele was constructed into pGMB152. Two DNA fragments (1083-bp *hsdM*12 and 1103-bp *hsdM*34) flanking the *hsdM* gene were amplified by PCR using the primers shown in Table 2. The in vitro-modified allele of *hsdM* 12-34 (Δ*hsdM*) was amplified using splicing by overlapping Extension PCR using primers *hsdM*12-F and *hsdM*34-R, because the primer *hsdM* 34-F was the reverse complement of the primer *hsdM* 12-R [5]. The amplified fragment was sub-cloned into the *Sal* I site of pMD20-T. A kanamycin-resistant gene cassette (*Km*^*R*^) was inserted at the *Xho* I in the middle of the fragment, with a *Sal* I site at each end [3, 8]. Subsequently, the DNA fragment between the two *Sal* I sites in the plasmid pMD20T-Δ*hsdM* was introduced into the *Sal* I cloning site of suicide plasmid pGMB152 to construct recombinant suicide plasmid pGMB152-Δ*hsdM*.

**Table 2:** Primers used in this study (enzyme sites are shown in lower case)

Fragments amplified	Primers	Primer sequences (5′-3′)	Amplicon size (bp)	Note
*hsdM*-*λ*	*hsdM*-*λ*	CTGCTGACCGAAATGCTCGAACC GTTCCAGGGCAAAATTTATGACC CCTGCTGCtgtgtaggctggagctgcttcg		
	*hsdM*-*λ*	TCGGCTTCGCCGCTGGTGTTGGA GCTCATCGAGCCGTTCGCCAGCA CAAAACCGcatatgaatatcctccttag		
*hsdM*12	*hsdM*12-F	aagtcgacctcgagATGCGGGTTCGGTTT GTTTG	1083	*Sal* I, *Xho* I
	*hsdM*12-R	TTCCTGATTGAGTTCATCATGGCC TGAAAAGACAATCCCACTCAATG		
*hsdM*34	*hsdM*34-F	CATTGAGTGGGATTGTCTTTTCAG GCCATGATGAACTCAATCAGGAA	1103	*Sal* I
	*hsdM*34-R	aagtcgacTGGTCCGAGCCGGATAAATG		
Δ*hsdM*-YZ	Δ*hsdM*-YZ-F	TGACCACCACCAATGCTACC	1920 (wt) 301 (Δ)	
	Δ*hsdM*-YZ-R	GTGGAACTGCTGGATGTGGA		
*flhA*-*λ*	*flhA*-*λ*-F	CCTTTAATATTGCGCTATCGATCATG GTGCTGCTGGTGGCGATGTTTACCC AGAtgtgtaggctggagctgcttcg		
	*flhA*-*λ*-R	CCAGCGCCGGGATCTGGGCGACCAG GCCGTCGCCAATGGTCAGCAGGGTG TAGCcatatgaatatcctccttag		
*flhA*(P125109)	*flhA*-F	AAgtcgac ctcgag CGGTAAGTCGCTACAGCCAA	2349	*Sal* I, *Xho* I
	*flhA*-R	AAgtcgac agatct CCTGAG ACACGATCCAAC		*Sal* I
flgK-*λ*	flgK-*λ*-F	TATTCTGGCGCAGGCAAACAGT ACGTTAGGGGCTGGCGGCTGGA TAGGTAATGGtgtgtaggctggagctgcttcg		
	flgK-*λ*-R	TGCGCCACGCTGGAGCCAATCGCGA TATTGACCTGTTTATCCTGATCGCGC AGAcatatgaatatcctccttag		
*flgK*(P125109)	*flgK*-F	AAcccggg CTCGAGGCTGAA AAGTATGCGCGAGG	2240	*Sma* I, *Xho* I
	*flgK*-R	cccggg AAGTTATCGCTGTCG CCGGTAT		*Sma* I

### Blue–White Selection for White Double-Crossover from Blue Single-Crossover Bacteria

Following a previous protocol [8], the plasmid pGMB152-Δ*hsdM* was electroporated into *E. coli* χ7213, which is diaminopimelic acid (DAP) dependent and kanamycin resistant. Blue colonies were selected on LB agar with 40 μg/ml X-gal, 1 % DAP, and three antibiotics (kanamycin, ampicillin, and streptomycin). Conjugation was then carried out between a donor of blue *E. coli* χ7213 (pGMB152-Δ*hsdM*) and a recipient of *S.* Pullorum *hsdM::Cm*^*R*^. Equal volumes of 24-h LB cultures from the donor and recipient were mixed and spotted immediately onto a nylon filter on an LB plate [8, 9]. After 36 h of conjugation at 37 °C, the filter was washed with 10 mM MgSO_4_ and the bacteria were plated onto LB plates containing X-gal and the three antibiotics (kanamycin, ampicillin, and streptomycin). The blue colonies presumably resulted from a single-crossover event where the suicide plasmid pGMB152 was integrated into the chromosome at *hsdM*. A random single colony was purified and sub-cultured 4–5 times in liquid LB with 10 % sucrose and without NaCl [7, 16]. Bacteria were then plated onto LB agar plates and screened for white colonies without chloramphenicol resistance to confirm the presence of plasmid excised double-crossover events. All white colonies were detected again with chloramphenicol selection: the double-crossover deletants should be susceptible to chloramphenicol because *Cm*^*R*^ gene was replaced by in vitro-modified allele.

### Confirmation of Deletants by PCR

The chromosomal duplication was segregated by homologous recombination between the flanking direct repeats, ultimately leaving one copy of the gene on the chromosome: either the wild-type copy or the deletant copy, and the suicide plasmid pGMB152 were lost simultaneously. The white colonies were further identified by PCR using primers Δ*hsdM*-YZ-F and Δ*hsdM*-YZ-R to distinguish the deletants from the wild-type bacteria.

### Two-Step Knockin of the *flhA* and Site-Directed Mutagenesis of *flgk*

Following the process of scarless–markerless knockout of *hsdM* gene, all primers were in the Table 2. The λ-red method was used to respectively insert the *Cm*^*R*^ gene into pseudogene *flhA* in which 23 bp from 601 to 623 was lost, using primers *flhA*-*λ*-F/R, and into the *flgK* gene in which a nucleotide GAA at 373 was changed to TAA to make a termination codon, using primers *flgK*-*λ*-F/R in *S*. *pullorum*. We then cloned a real gene from *S. enteritidis* P125109 to replace the pseudogene in *S. pullorum* to construct an in vitro-modified allele. Using the blue/white screen strategy in addition to counter selection screening (*sacB* gene), the mutants with a knocked-in *flhA* gene and mutated *flgk* gene were screened. The two genes were amplified by PCR and sequenced for further confirmation.

## Results and Discussion

**Fig. 1:**
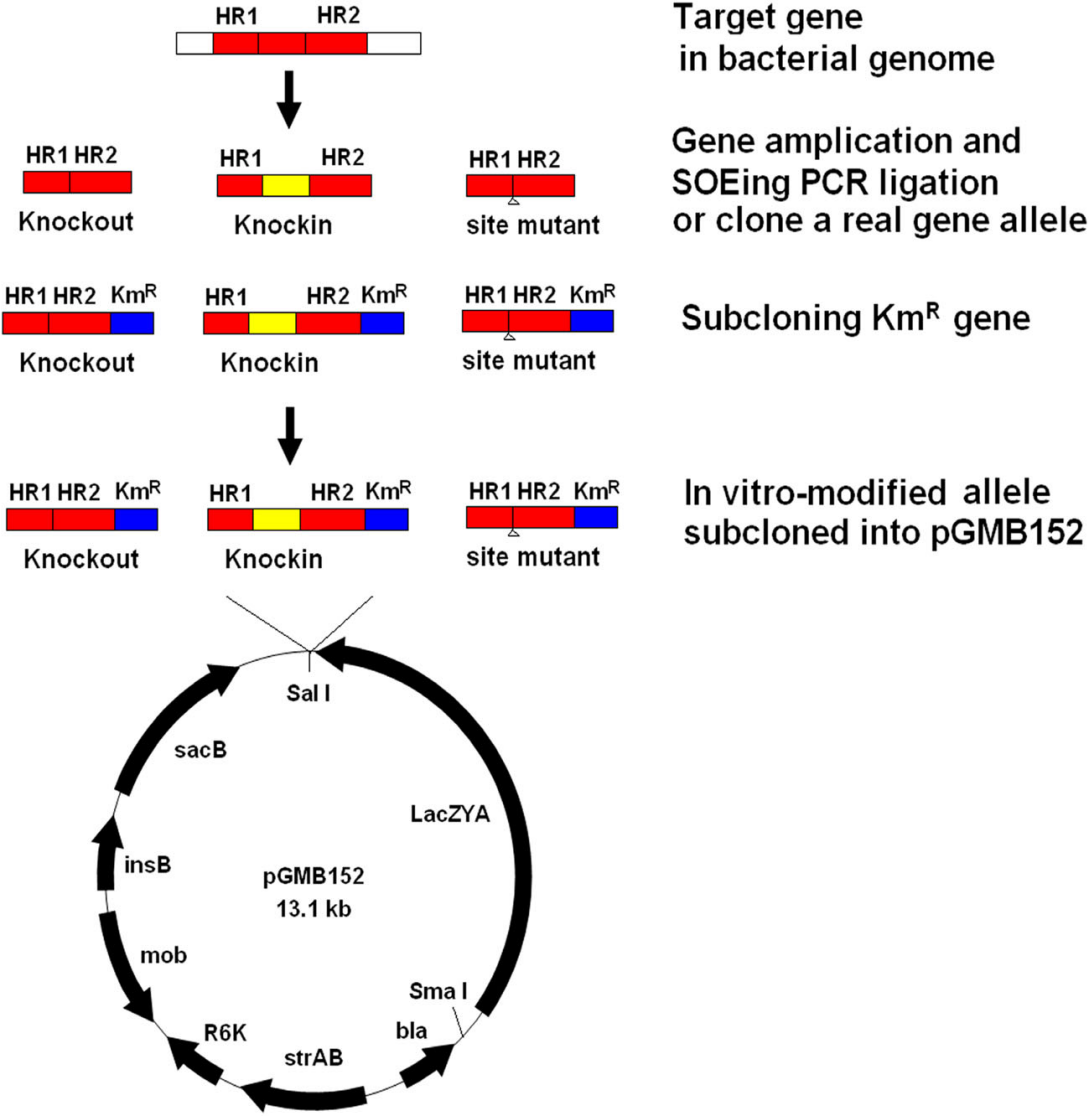
Construction of an in vitro-modified allele

After construction of the recombinant suicide plasmid pGMB152-Δ*hsdM/Km*^*R*^ and conjugation, only *S. pullorum hsdM::Cm*^*R*^ pGMB152-Δ*hsdM/Km*^*R*^ grew as single-crossover recombinants on LB plates with the antibiotics (chloramphenicol, streptomycin, ampicillin, kanamycin) and without DAP, all colonies were blue (Fig. 1 left-hand panel).

When blue *S. pullorum hsdM::Cm*^*R*^ pGMB152-Δ*hsdM/Km*^*R*^ was sub-cultured in liquid media with sucrose and without NaCl, a few bacteria turned white, indicating that plasmid excision double-crossover had occurred (Fig. 1 right-hand panel).

**Fig. 2:**
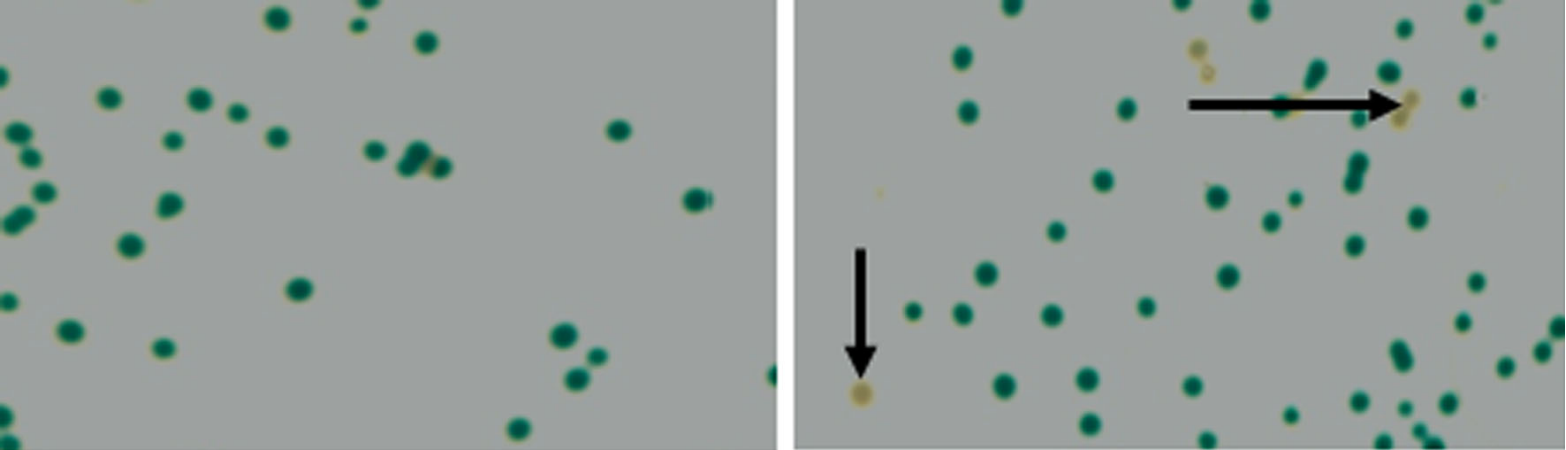
Blue–white selection. *Left-hand panel* shows *blue* colonies resulting from a single-crossover event, and the *right-hand panel* shows the white colonies (possible *hsdM* deletants) from a double-crossover event (example of *hsdM*) (Color figure online)

The theoretical probability of finding deletants among the white colonies would be 50 %, because the two gene arms have an identical chance to exchange with the homologous sequence in the target genome. Approximately 10 % of all white colonies were found to be deletants based on incomplete statistics. White deletants were confirmed by PCR with Δ*hsdM*-YZ primers based on their amplicon size and antibiotic resistances; wild-type colonies were excluded (Fig. 2). The amplicon size should be 301 bp for the white deletants, 1920 bp for white wild-type strain, and both 1920 bp and 301 bp for the blue clones as a control. The amplicon sizes were consistent with the expected results.

**Fig. 3:**
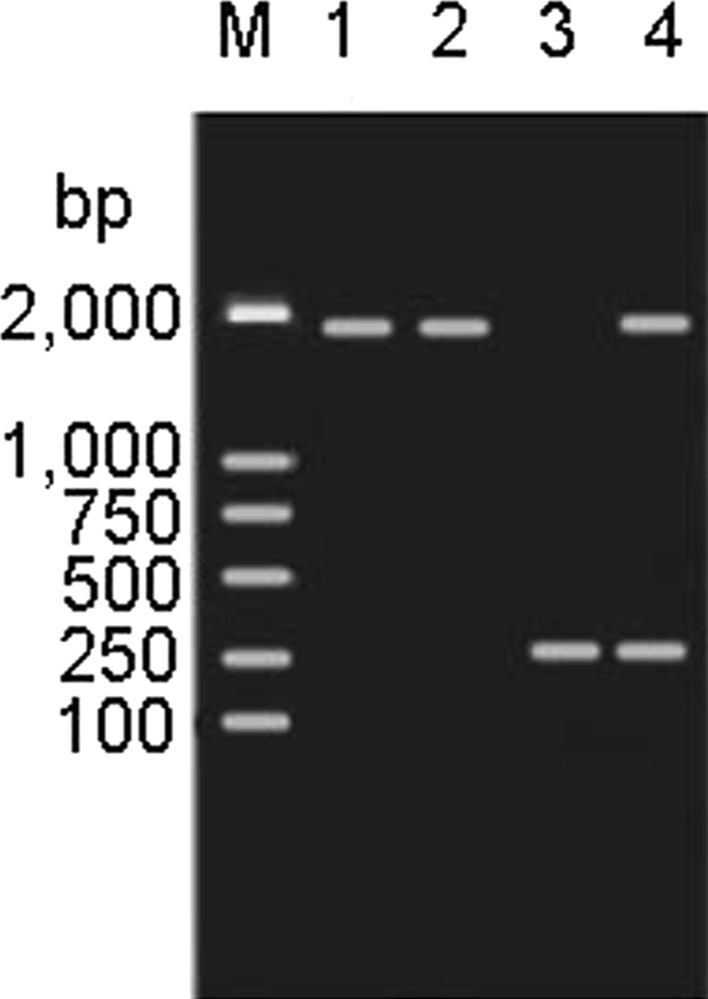
PCR identification of *hsdM* deletants from *white* colonies. M: DNA Marker DL2000; lane1: S06004 as a control; lane2: White colony 1(wild type S06004); lane3: White colony 2 (S06004Δ*hsdM*); lane4: Blue colony(S06004(pGMB152-Δ*hsdM*)).

**Fig. 4:**
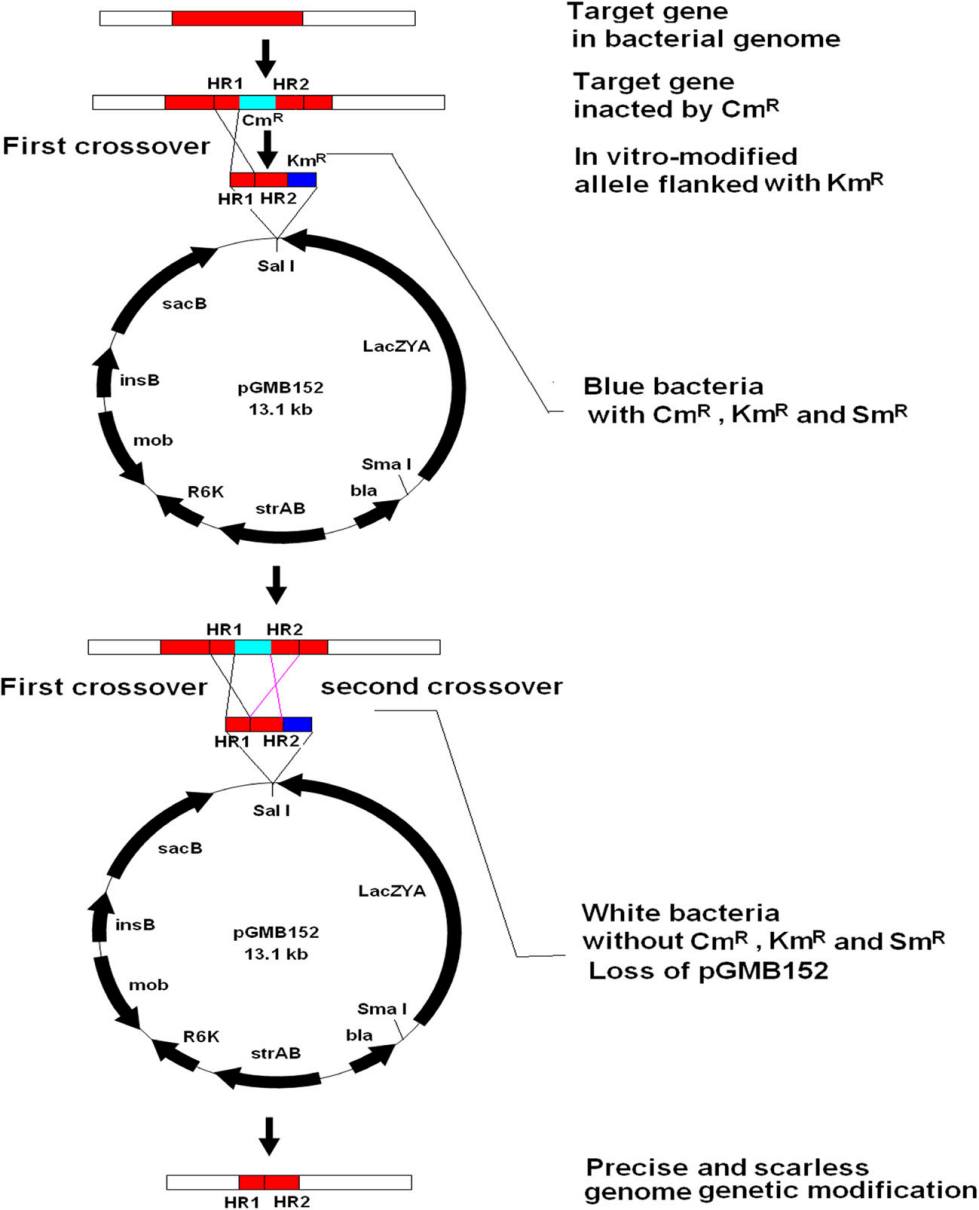
The process of two-step scarless–markerless genome genetic modification based on suicide plasmid pGMB152

Gene knockin and site-directed mutagenesis might be more difficult than gene knockout. We believed that improved plasmid could also be applied successfully for scarless–markerless gene knockin to repair the *flhB* and *flgK* pseudogenes in *S.pullorum*, following the process summarized in Fig. 3. After repair, the two genes, *flhA* and *flgK*, were amplified and sequenced for further confirmation. The results showed that the original pseudogenes were replaced by real genes. We successfully generated the knockin of the real *flhA* gene using 23 bp from 601 to 623 and performed site-directed mutagenesis of *flgK* to change nucleotide 373 from TAA, which is a termination codon, to GAA in S. *pullorum* (Fig. 4).

In this study, we found that sucrose-counter selection was relatively inefficient, as indicated by the presence of large numbers of blue colonies among the transformants (Fig. 1 right-hand panel) in *S.pullorum*, possibly because of host adaptation. This further confirmed that double-crossover events occurred rarely in bacteria following the traditional method [7, 16]. The results indicated that we could select random single-crossover recombinant bacteria without needing to reassess their sucrose sensitivity [8].

The larger size of vector pGMB152 might make molecular manipulations more difficult. To overcome this problem, an antibiotic resistance gene was cloned in one flank of the in vitro-modified allele for easy selection of recombinants, such as the kanamycin resistance gene in this study (Fig. 1).

This study is the first report of a suicide plasmid comprising the complete *lacZ* gene that allows visual selection of double-crossover mutants. Although pGMB152 had only one modification, i.e., the entire *lacZYA* operon, compared with pGMB151, it was more efficient and less time consuming in producing allelic exchanges of gram-negative bacteria because of its color discrimination. Thus, the improved suicide plasmid, pGMB152, will be helpful for precise and scarless–markerless genome genetic modification.
